# Identification of a novel functional JAK1 S646P mutation in acute lymphoblastic leukemia

**DOI:** 10.18632/oncotarget.16670

**Published:** 2017-03-29

**Authors:** Qian Li, Botao Li, Liangding Hu, Hongmei Ning, Min Jiang, Danhong Wang, Tingting Liu, Bin Zhang, Hu Chen

**Affiliations:** ^1^ Department of Hematopoietic Stem Cell Transplantation, Affiliated Hospital of the Academy of Military Medical Sciences, Beijing, China; ^2^ Cell and Gene Therapy Center, Affiliated Hospital of the Academy of Military Medical Sciences, Beijing, China

**Keywords:** acute lymphoblastic leukemia, whole-exome sequencing, mutation, JAK1, ruxolitinib

## Abstract

The survival rate of childhood acute lymphoblastic leukemia (ALL) is approaching 90%, while the prognosis of adults remains poor due to the limited therapeutic approaches. In order to identify new targets for ALL, we performed whole-exome sequencing on four adults with B-ALL and discovered a somatic *JAK1* S646P mutation. Sanger sequencing of *JAK1* was conducted on 53 ALL patients, and two cases exhibited A639G and P960S mutations separately. Functional studies demonstrated that only *JAK1* S646P mutation could activate multiple signaling pathways, drive cytokine-independent cell growth, and promote proliferation of malignant cells in nude mice. Moreover, a high sensitivity to the JAK1/2 inhibitor ruxolitinib was observed in S646P mutant model. Exploration in a total of 209 ALL cases showed that JAK1 mutations occur at a frequency of 10.5% in T-ALL (2/19) and 1.6% in B-ALL (3/190). Collectively, our results suggested that *JAK1* S646P is an activating mutation *in vitro* and *in vivo*. JAK-STAT pathway might represent a promising therapeutic target for ALL.

## INTRODUCTION

Acute lymphoblastic leukemia (ALL) is a malignant clonal disorder caused by the uncontrolled proliferation of immature lymphoblastic cells in bone marrow, which originate from the abnormal development of hematopoietic stem cells or lymphoid progenitor cells, and may be of B- or T- lymphoid lineage (B-ALL or T-ALL) [1]. ALL is seen in both children and adults, but with a peak at the age of 2–5 years [2]. Given the considerable progress in risk stratification and chemotherapy, the long-term survival rate of childhood ALL has improved to approximately 90% [3]. Although the overall survival of adult ALL improved to 30–40% after adaptation of pediatric protocols, the prognosis remains poor due to the unsatisfactory response to chemotherapy [4].

Genetic alterations are vital events in leukemogenesis, and are closely related to the overall prognosis and relapse risk. Majority of ALL cases exhibit recurrent genetic alterations that are detectable by karyotyping, FISH or molecular techniques [5]. Current risk-stratification is mostly based on the presence and types of genetic abnormalities. BCR-ABL1 is the most common molecular abnormality in adult ALL and associated with poor outcome; by contrast, hyperdiplody and ETV6-RUNX1/TEL-AML1 (both associated with favorable outcome) are more common in childhood ALL, which may be partly responsible for the better outcome in children than that in adults [6]. However, current therapies that target specific genetic alterations remain insufficient, except for tyrosine kinase inhibitors (TKIs) such as imatinib in the treatment for BCR-ABL1-positive leukemia [7]. Therefore, the discovery of novel biological targets and agents is of great interest.

In order to identify novel mutations and therapeutic targets in ALL, we conducted whole-exome sequencing (WES) on four adult B-ALL patients without any known cytogenetic and molecular abnormality at diagnosis. In the course of screening, we identified and successfully validated a somatic *JAK1* S646P mutation. Sanger sequencing of *JAK1* gene on additional 53 ALL cases discovered A639G and P960S mutations. Among them, only S646P mutation was proved to be an activating mutation *in vitro* and *in vivo*. Moreover, the S646P mutant model exhibited an increased sensitivity to ruxolitinib, suggesting that it may act as a potential therapeutic target. The prevalence and clinical relevance of *JAK1* mutations were investigated in a total of 209 ALL cases, showed that *JAK1* mutations occur at a frequency of approximately 10.5% in T-ALL and 1.6% in B-ALL.

## RESULTS

### Identification and validation of *JAK1* mutations in patients with ALL

**Figure 1:**
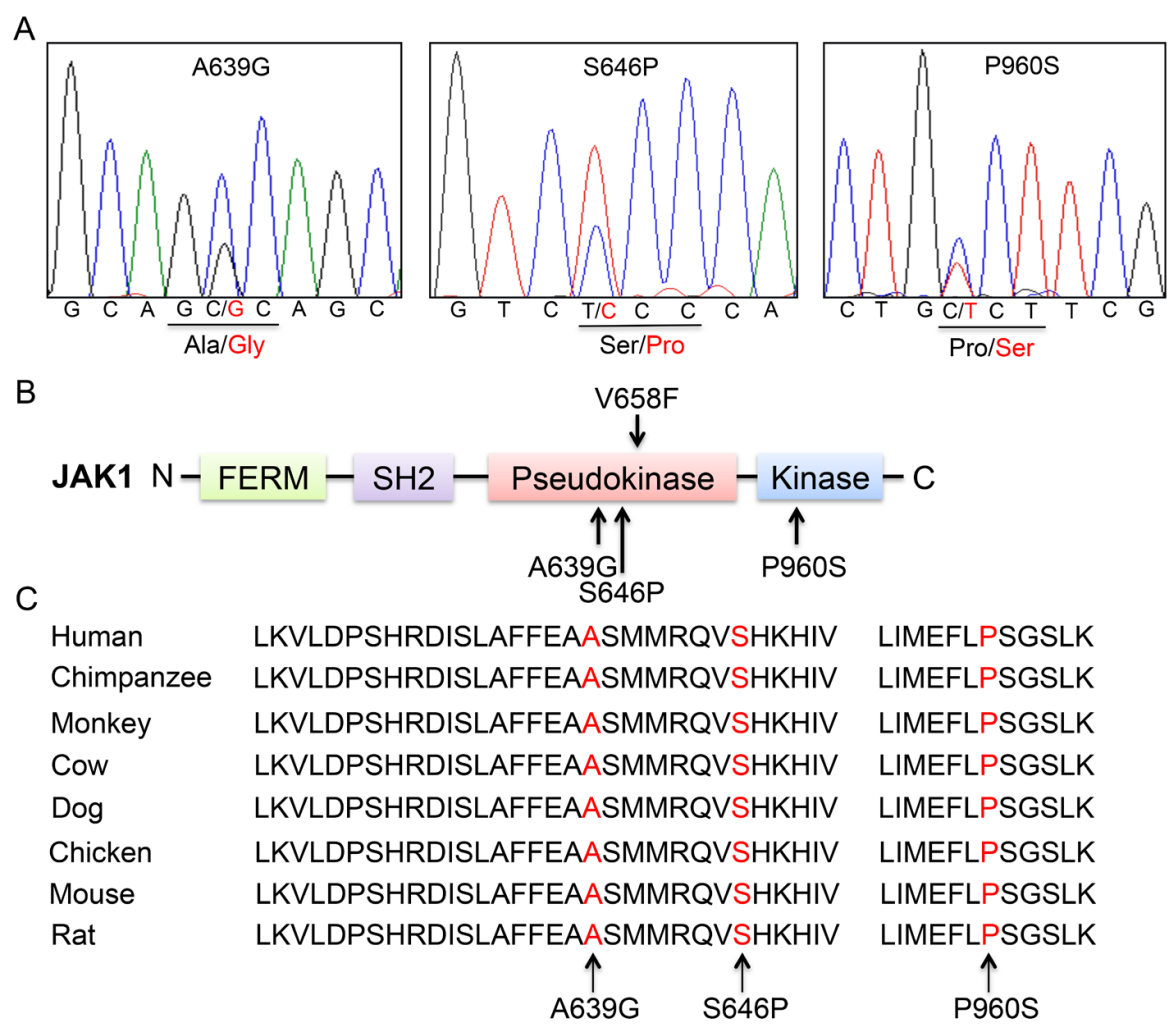
Somatic non-synonymous mutations in conserved residues of JAK1 (**A**) Representative electropherograms of identified *JAK1* mutations from 3 patients with ALL. Heterozygous mutations are indicated by double peaks. (**B**) Schematic diagram of JAK1 protein structure and location of affected residues. Somatic mutations are indicated by arrows. (**C**) Alignment of amino acid sequences of JAK1 residues.

WES was successfully performed on newly diagnosed (blasts > 80%) and paired remission (blasts < 5%) bone marrow samples from four adults with B-ALL, who were not diagnosed with any known cytogenetic and molecular abnormality through routine laboratory examinations. The clinical characteristics of the four patients were listed in Table 1. To ensure the reliability and accuracy of the sequencing data, the average coverage of each base in the targeted regions was 50-fold; 80% of the bases were represented at least 10 times. A total of 317 non-synonymous mutations and 27 nonsense mutations were identified in 337 genes (listed in Supplementary Table 2). 29 non-synonymous mutations in 24 genes were further validated by Sanger sequencing (listed in Supplementary Table 1). Somatic heterozygous *JAK1* S646P mutation (located at exon 14), *DOT1L* F131L and E134K mutations (located at exon 5) were successfully validated. We then sequenced *JAK1* exon 14 and *DOT1L* exon 5 in additional 53 newly diagnosed ALL patients (B-ALL, *n* = 46; T-ALL, *n* = 7) using Sanger sequencing. No additional *DOT1L* mutation was identified, but a novel *JAK1* A639G mutation was found in one sample. Subsequently, all 25 exons of *JAK1* were analyzed in these 53 cases using Sanger sequencing, and the P960S mutation was identified in another sample. Together, all of the three identified *JAK1* mutations were heterozygous (Figure 1A). Among them, S646P and A639G mutations are newly discovered, and located at exon 14 in the pseudokinase domain, while P960S mutation (COSM4990582), which is located at exon 21 in the kinase domain, has been described in cutaneous squamous cell carcinoma [8] (Figure 1B).

**Table 1:** Clinical characteristics of the four patients analyzed by WES

Patient	Gender	Age	Lineage	Blasts (%) (diagnosis/remission)	Cytogenetic abnormalities	Fusion Gene
1	M	22	B-ALL	81.0/5.0	-	-
2	M	15	B-ALL	95.5/3.0	-	-
3	M	22	B-ALL	92.0/2.5	-	-
4	F	40	B-ALL	96.2/2.5	-	-

Abbreviations: WES, whole-exome sequencing; ALL, acute lymphoblastic leukemia; M, male; F, female.

Alignment of *JAK1* gene sequences from different species revealed that the mutant residues are highly conserved throughout vertebrate evolution (Figure 1C). These data demonstrated that the identified mutations are likely to play important roles in the structure and function of JAK1 protein.

### *JAK1* S646P mutation induces constitutive activation of JAK-STAT and MAPK-ERK signaling pathways

**Figure 2:**
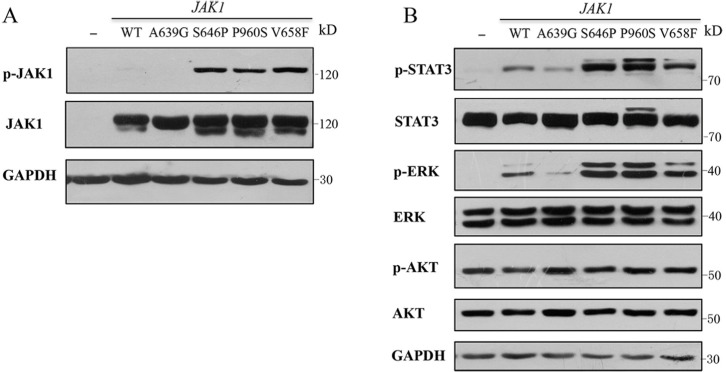
*JAK1* S646P mutation induces constitutive activation of JAK-STAT and MAPK-ERK signaling pathways (**A**) Autophosphorylation of JAK1 protein in HEK293V cells transiently transfected with wild-type (WT) or a mutant of *JAK1* (A639G, S646P, P960S and V658F). (**B**) Phosphorylation of STAT3, ERK1/2 and AKT in HEK293V cells. Protein lysates of HEK293V cells transiently transfected with WT *JAK1* or indicated mutants were analyzed by Western blot using specific antibodies shown. GAPDH was used as loading control. Similar results were obtained in 3 independent experiments.

Given that HEK293V lack some endogenous cytokine receptors, such as IL-2R, IL-9Rα and common γ chain [9], cells that express gain-of-function mutations could induce constitutive activation of signaling pathways independent of cytokines and receptors, such as *JAK2* V617F and its homologous mutant of *JAK1* V658F [10]. To examine the effects of the identified mutations on signaling pathways, wild type (WT) or a mutant of *JAK1* (A639G, S646P and P960S) was introduced transiently into HEK293V cells, autophosphorylation of JAK1 and phosphorylation of STAT3, ERK1/2 and AKT were compared basally, and *JAK1* V658F mutation was used as a positive control. The data showed that expression of S646P and P960S mutations resulted in autophosphorylation of JAK1 (Figure 2A), and increased constitutive activation of STAT3 and ERK proteins, consistent with the positive control of V658F mutant. While WT and A639G groups exhibited no autophosphorylation of JAK1 and weak phosphorylation of STAT3 and ERK proteins (Figure 2B). No obvious difference was observed in AKT pathway among all groups. These data indicate that *JAK1* S646P mutation can constitutively activate JAK1-STAT3 and MAPK-ERK pathways.

### *JAK1* S646P mutation drives cytokine-independent growth of BaF3 cells

**Figure 3:**
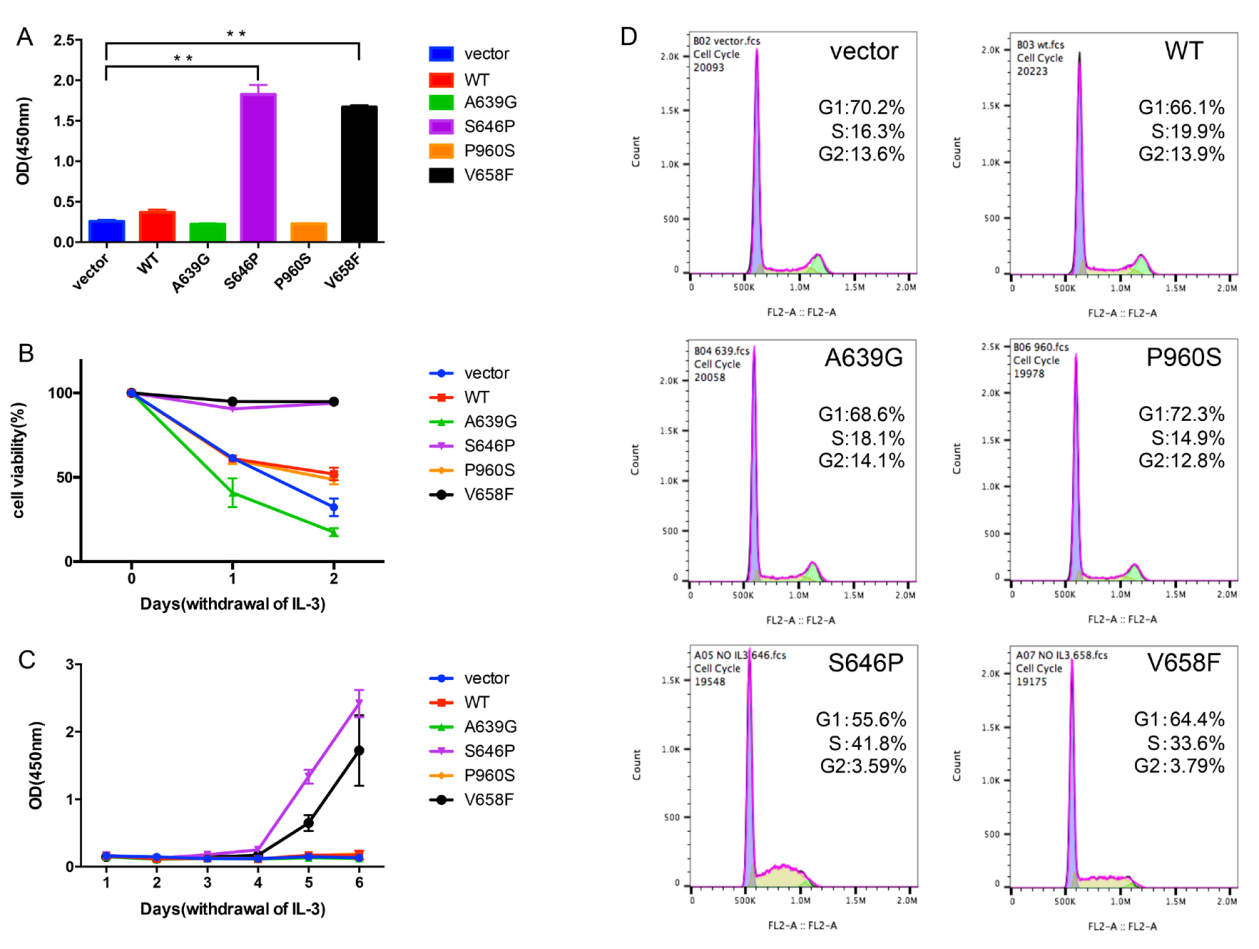
*JAK1* S646P mutation drives IL-3-independent growth of BaF3 cells (**A**) The proliferation of transduced BaF3 cells after withdrawing IL-3 for 3 days was assayed by CCK-8. Data are expressed as mean ± s.d of at least 3 independent experiments. **P < 0.01. (**B**) The viability of transduced BaF3 cells after withdrawing IL-3 for 48 h was determined by trypan blue exclusion method at the indicated time points. (**C**) The survival curves of transduced BaF3 cells cultured in the absence of IL-3 for 6 days were determined by CCK-8. (**D**) Cell cycle histograms of transduced BaF3 cells were assayed by flow cytometry. The proportion of G1 or S/G2 phase is expressed as percentage of total cells.

To explore the transforming capability of the identified mutations, we used a cytokine independent growth assay with the murine pro B-cell BaF3 cell line. BaF3 cells normally depend on IL-3 for growth, but can grow in the absence of IL-3 when expressing with an oncogene, such as BCR-ABL1 fusion gene [11] and *JAK2* V617F mutation [12]. We transduced BaF3 cells using lentiviral vector expressing WT or mutants of *JAK1* and selected for GFP-positive cells using puromycin (1 μg/ml). After withdrawing IL-3 for 3 days, BaF3 cells with S646P and V658F mutations could still grow, while other groups almost had no growth (Figure 3A).Meanwhile, we also observed that BaF3 cells with S646P and V658F mutations were resistant to IL-3 withdrawal-induced apoptosis, but cells with vector, WT or other mutants (A639G and P960S) underwent death in different degrees (Figure 3B). Cell survival curves also showed that only S646P and V658F mutations conferred the IL-3-independent growth of BaF3 cells (Figure 3C). Moreover, although BaF3 cells with S646P and V658F mutations were cultured without IL-3 in following experiments, they demonstrated an elevated percentage of S/G2 phase compared with other groups maintained with IL-3 (Figure 3D). These data suggest that both of *JAK1* S646P and V658F mutations are gain-of-function.

We also studied the effects of *JAK1* mutations on human B-ALL NALM-6 cell line, and observed that NALM-6 cells transduced with S646P mutation had an increased percentage of S/G2 phase relative to other groups (Supplementary Figure 1), further confirming the promoting effect on cancer cell proliferation of *JAK1* S646P mutation.

### *JAK1* S646P mutation leads to malignant proliferation of BaF3 cells in nude mice

**Figure 4:**
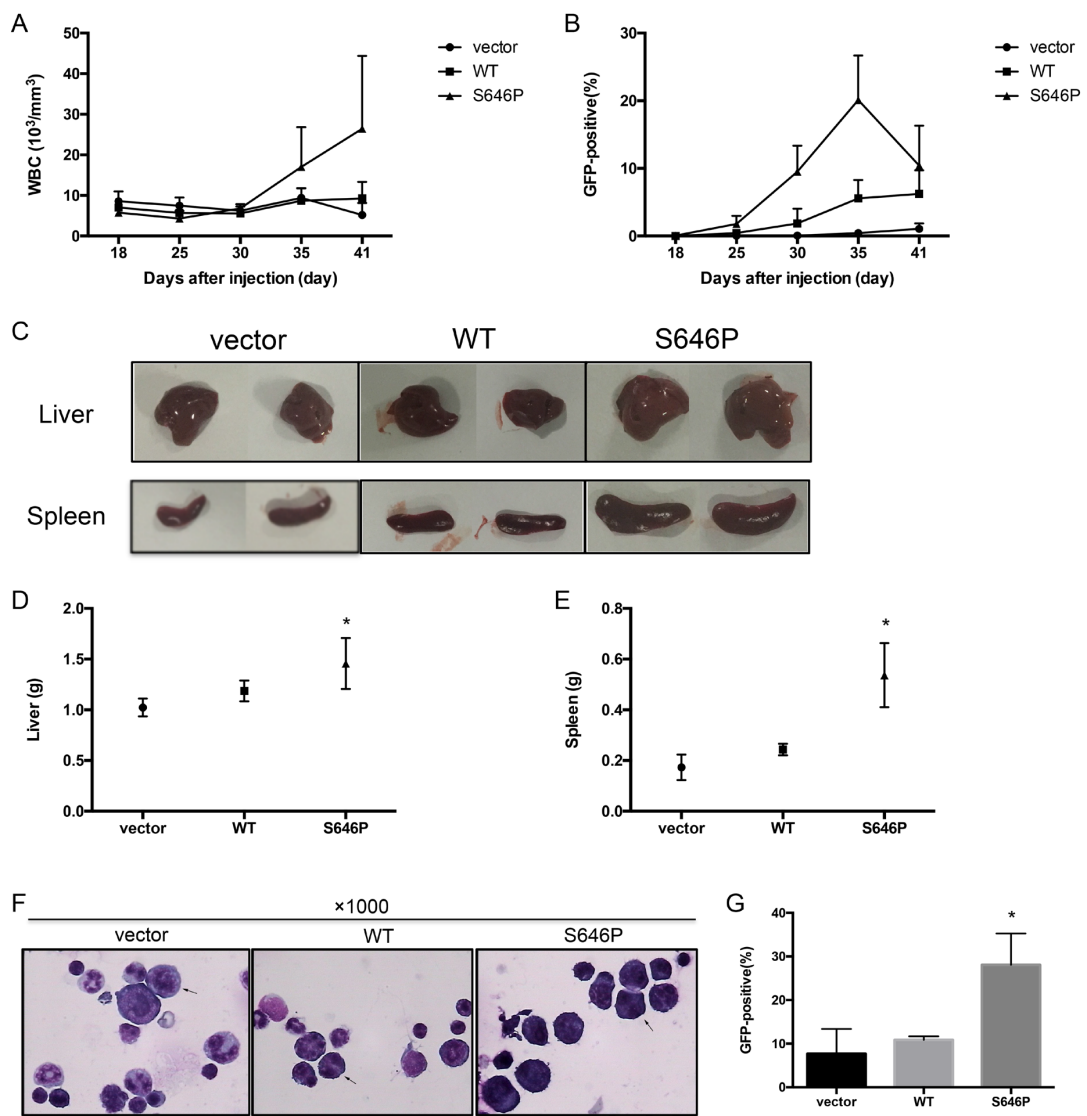
*JAK1* S646P mutation induces malignant proliferation of BaF3 cells in nude mice 2 × 10^6^ BaF3 cells transduced with vector, WT *JAK1*, or S646P mutant were intravenously inoculated into nude mice. (**A**) WBC counts were monitored at the indicated time points. (**B**) GFP-positive cells in peripheral blood were monitored by flow cytometry. (**C**) At the time of sacrifice, the morphological changes of livers and spleens were photographed. (**D**, **E**) The weights of livers (D) and spleens (E) were recorded. Data are expressed as the mean± s.d. *P < 0.05. (**F**) The bone marrow smears were stained with Wright and photographed (magnification: ×1000). Representative abnormal cells were marked with arrows. (**G**) The percentage of GFP-positive cells in bone marrow at the time of sacrificed was detected by flow cytometry. Data are expressed as the mean ± s.d. *P < 0.05.

To explore the oncogenic potential of BaF3 cells with *JAK1* S646P mutation *in vivo*, we examined whether the intravenous inoculation of transduced BaF3 cells into nude mice could induce malignant proliferation. 2 × 10^6^ BaF3 cells transduced with vector, WT *JAK1*, or S646P mutation were intravenously inoculated (tail vein) into nude mice. During the weekly monitoring of peripheral blood, the BaF3/S646P mice exhibited statistically significant elevated white blood cells (WBC) counts (Figure 4A) and GFP-positive cells infiltration (Figure 4B) after 30 days post-inoculation. At the time of death, the inoculation of BaF3/S646P cells induced enlargement of liver and spleen relative to vector and WT controls (Figure 4C). The average weight of liver in BaF3/S646P cell-inoculated mice increased by about 1.2 to 1.4 fold (1.46 ± 0.25 versus 1.19 ± 0.1 g for BaF3/WT and 1.02 ± 0.09 g for BaF3/vector, *P* < 0.05) (Figure 4D), and the average weight of spleen in BaF3/S646P mice increased by about 2 to 3 fold (0.54 ± 0.13 versus 0.24 ± 0.02 g for BaF3/WT and 0.17 ± 0.05 g for BaF3/vector, *P* < 0.05) (Figure 4E). Histologic analysis of bone marrow smears revealed increased abnormal cells in BaF3/S646P mice relative to control groups (Figure 4F). The percentage of GFP-positive cells in bone marrow of BaF3/S646P mice was also higher than that in vector and WT control groups (Figure 4G). Therefore, we consider that the over expression of *JAK1* S646P mutation could promote malignant proliferation of BaF3 cells in nude mice.

### *JAK1* S646P mutation exhibits increased sensitivity to JAK1/2 inhibitor

**Figure 5:**
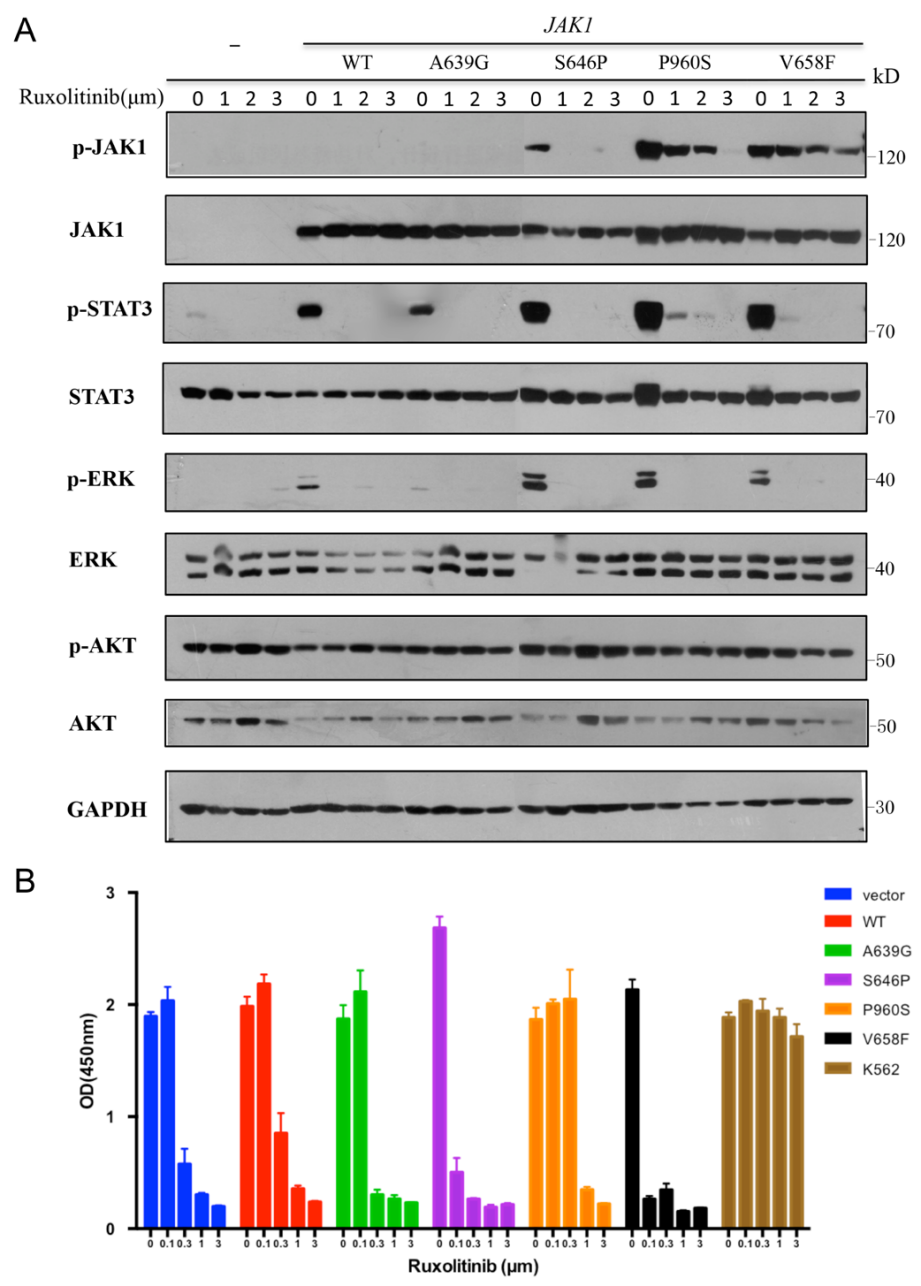
*JAK1* S646P mutation exhibits increased sensitivity to JAK1/2 inhibitor (**A**) Transiently transfected HEK293V cells were treated with increasing concentrations of ruxolitinib for 24 h. Cell lysates were immunoblotted with specific antibodies shown. GAPDH was used as loading control. (**B**) Transduced BaF3 cells were treated with increasing concentrations of ruxolitinib for 48 h. Cell proliferation was determined by CCK-8; K562 cells were used as negative control. Results represent the mean ± s.d. of 3 independent experiments.

To detect the sensitivity of *JAK1* mutations to inhibitor, we examined whether ruxolitinib as an inhibitor for JAK1/2 could inhibit the activation of *JAK1* mutants. As shown in Figure 5A, treatment with increasing concentrations of ruxolitinib inhibited the autophosphorylation of JAK1 and constitutive activation of STAT3 and ERK1/2.

Furthermore, we treated transduced BaF3 cells with increasing concentrations of ruxolitinib or solvent (DMSO) and assayed their proliferation. Except for autonomous BaF3 cells with S646P or V658F mutation, the rest of groups were cultured with IL-3. Chronic myeloid leukemia cell line K562 with BCR-ABL1 fusion gene was employed as a negative control due to its lack of sensitivity to JAK inhibitors [13]. Figure 5B showed that all transduced BaF3 cells were inhibited by high concentrations of ruxolitinib (> 0.1 μm), while BaF3 cells with S646P and V658F mutations presented increased sensitivity to ruxolitinib, as at the concentration of 0.1μm, their cell proliferation have been significantly inhibited relative to other groups.

### Prevalence and clinical relevance of *JAK1* mutations

Since the identified *JAK1* mutations were focused in exon 14 and 21, we sequenced the two exons in additional 152 ALL patients (B-ALL, *n* = 140; T-ALL, *n* = 12) to determine the prevalence of *JAK1* mutations. Two patients with B-ALL were discovered with Y652H and N973K mutations separately. Y652H has been described to be gain-of-function in T-ALL [14, 15], and N973K could be searched in dbSNP (rs34680086). Together, a cohort of 209 ALL patients were investigated, which is not restricted to a particular genetic subgroup (listed in Supplementary Table 3), and the prevalence of *JAK1* mutations were found at a frequency of approximately 2.4% in ALL (5/209), especially more common in T-ALL (2/19) than in B-ALL (3/190) (10.5% versus 1.6%, *P* < 0.05) (Table 2).

**Table 2:** The occurrence of *JAK1* mutations in different subtypes of ALL

Subtypes	Events/Patients (Frequency)		
	Adults	Children	Total
B-ALL	2/109 (1.8%)	1/81 (1.2%)	3/190 (1.6%)*
T-ALL	2/19 (10.5%)	0	2/19 (10.5%)*
Total	4/128 (3.1%)^#^	1/81 (1.2%)^#^	5/209 (2.4%)

Notes: The comparisons of occurrence of *JAK1* mutations in B-ALL and T-ALL or adults and children were performed using chi-square test, and *p* values < 0.05 were considered statistically significant. *P < 0.05; ^#^*P* > 0.05.

The characteristics of patients with *JAK1* mutations were investigated and listed in Table 3. We observed that four mutation-positive patients ever relapsed and resisted to chemotherapy. Patient 1 with S646P mutation relapsed twice, and even presented the same mutation in relapsed blasts, indicating that this mutation may be associated with his relapse. Patient 3 with P960S mutation relapsed early after induction therapy, and resisted to multiple chemotherapies, even developed extramedullary infiltration in right eye. Patient 4 with Y652H mutation harbored a complex karyotype (48, XY, +5, del (14)(q24), +19) at diagnosis. He relapsed extramedullarily at 3 years post-HSCT, diagnosed as B-cell lymphoblastic lymphoma encroached on double orbits and nasal cavity. N973K mutation was identified in a 3-year-old child (patient 5), who had a complex karyotype and the TCF3-PBX1 fusion gene known to be associated with a high relapse risk [16]. He also relapsed early after a standard induction therapy. These data indicated that *JAK1* S646P mutation might be associated with the patient's relapse, especially in the absence of other adverse prognostic factors. In addition, *JAK1* mutations may cooperate with other genetic aberrations to contribute to the dismal clinical course.

**Table 3:** Characteristics of ALL patients with *JAK1* mutations

P	G	Age	Lineage	WBC (× 10^9^/L)	Fusion Gene	Complex karyotype	AA substitution	Relapse
1	M	15	B-ALL	18.55	-	-	Ser646Pro	+
2	M	32	T-ALL	25.2	-	-	Ala639Gly	-
3	M	42	T-ALL	320.29	-	-	Pro960Ser	+
4	M	17	B-ALL	11	-	+	Tyr652His	+
5	M	3	B-ALL	45.95	TCF3-PBX1	+	Asn973Lys	+

Abbreviations: ALL, acute lymphoblastic leukemia; P, patient; G, gender; WBC, white blood cell; AA, amino acid; M, male.

## DISCUSSION

In the present study, we characterized a novel functional *JAK1* S646P mutation in ALL identified by WES, which was able to activate JAK-STAT and MAPK-ERK signaling pathways, and drive IL-3-independent growth of BaF3 cells. *In vivo*, the inoculation of BaF3 cells with *JAK1* S646P mutation induced malignant proliferation in nude mice. These data support that *JAK1* S646P mutation is an activating mutation in ALL. In addition, other genes known to be affected by somatic mutations in hematologic cancers were also found from the WES data (Supplementary Table 3), such as JAK3, CREBBP, BCOR, and so on. JAK3 mutations were initially identified in patients with acute megakaryocytic leukemia [17], and mainly found in T-ALL and NKT lymphoma [18, 19]. The deletion and sequence mutations of CREBBP, which encodes the transcriptional coactivator and histone acetyltransferase (HAT) CREB-binding protein (CBP), have been reported in 18.3% of ALL relapse cases, and the mutations may confer resistance to therapy [20, 21]. Besides, CREEB mutations also have been reported in 39% of DLBCL and 41% of follicular lymphoma (FL) patients [22]. BCOR, which possesses transcriptional activity, has been reported to be mutated in 4% of T-ALL [23] and 4.2% of MDS [24], and also associated with the disease progression.

JAK genes encode non-receptor tyrosine kinases, including 4 members, JAK1, JAK2, JAK3, and TYK2 [25]. A point mutation of *JAK2* V617F was found in more than 50% of patients with myeloproliferative neoplasms (MPNs) [26]. This mutation has shown to be gain-of-function *in vitro* and *in vivo* [27]. Previous studies demonstrated that *JAK1* V658F mutation is homologous to *JAK2* V617F, and able to constitutively activate JAK1-STAT3 pathway [10, 28, 29]. Both of V658F and S646P mutations are located in the pseudokinase domain of JAK1, and exhibit a similar function in the present study, indicating that S646P mutation may cause the constitutive activation of JAK1 kinase via disrupting the auto-inhibition as V658F. Similarly, *JAK1* A634D and R724H mutations described in T-ALL [30], and V623A mutation in AML [31], were also located in the pseudokinase domain and exhibited activating function *in vitro* proliferation assays. In addition, similar to our results in nude mice, BaF3 cells transduced with *JAK2* L611S mutation, which was reported in a child with ALL [32], has also been proposed to be of oncogenic ability to induce tumorigenesis in nude mice and prompt invasion into various organs [33].

The pseudokinase domain of JAK1 protein is analogous to the kinase domain, and functions as a negative regulator of kinase activity [34]. The available data support the view that the recurrent activating mutations are mostly located at the pseudokinase domain of JAKs [14, 29, 30], the pathogenic mechanism is predicted to interfere with auto-inhibition on the catalytic activity [30]. However, we provide the first evidence that not all mutations at pseudokinase domain are gain-of-function, such as *JAK1* A639G mutation, which did not induce the cytokine-independent cell growth and activation of downstream signaling pathways. This finding indicated that the molecular modeling and location of affected residues should be fully characterized. It is proposed that structural rearrangements in the regions involved in interdomain interactions between the pseudokinase and kinase domains or the FERM and SH2 domains, particularly in G-Loop, which participates in active tyrosine kinases [31], are likely to be gain-of-function [35]. In addition, it seems that the mutations at kinase domain have no transforming capability, since P960S mutation cannot drive cytokine-independent cell growth, similar to R879C described in T-ALL [30]. However, both of them showed increased activation of signaling pathways, suggesting that the cell transformation process is involved with other signals or molecules interacting with the pseudokinase domain.

Adverse prognostic factors in adult ALL consist of age (> 35 years), an elevated WBC count (> 30 × 10^9^/L in B-ALL and > 100 × 10^9^/L in T-ALL), and complex karyotype. Flex et al. reported *JAK1* mutations occurred at a frequency of 4.6% in ALL, also more commonly in T-ALL than in B-ALL. They provided evidence that *JAK1* mutations in adult T-ALL are associated with a poor response to therapy, frequent relapse and reduced overall survival [30]. In our study, we observed that four mutation-positive patients ever relapsed and resisted to chemotherapy. However, based on the available data, we cannot confirm whether *JAK1* mutation is a poor prognosis factor for ALL with the relatively small size of the study cohort. Larger cohorts are required to estimate the clinical relevance of such mutations in ALL. Furthermore, the dysfunction of *JAK1* mainly results in impaired lymphopoiesis [36], consistent with previous studies [29, 30, 37], we revealed that *JAK1* mutations occur at a frequency of approximately 2.4% in ALL, and more commonly in T-ALL than in B-ALL. This result can be explained by the opinion that activation of JAK1 and NOTCH1 pathways might cooperate in T-ALL pathogenesis and progression [23, 30], encouraging the studies on combination of several targeted therapies in refractory/relapse cases..

A JAK1/2 inhibitor, ruxolitinib, has been approved by FDA for the treatment of patients with intermediate or high-risk myelofibrosis and polycythemia vera [38, 39]. Recently, it was also tested in clinical trials for the treatment of Ph-like ALL, which was identified by abnormalities in the Ras and JAK/STAT pathways. We showed that all of *JAK1* mutations are sensitive to ruxolitinib, and S646P mutation exhibits an increased sensitivity, indicating that ruxolitinib could be valuable for patients with *JAK1* abnormalities. Combined multiple targeting drugs may be of more clinical benefit for ALL.

In summary, our work provides the first evidence that the newly identified *JAK1* S646P mutation is an activating mutation *in vitro* and *in vivo*, and highly sensitive to JAK inhibitor, encouraging studies aimed at testing the efficacy and side effects of ruxolitinib in the treatment for ALL. Moreover, studies on larger cohorts are required to estimate the clinical relevance and prognostic value of such mutations in ALL.

## MATERIALS AND METHODS

### Human subjects

The Institutional Review Board of the Affiliated Hospital of the Academy of Military Medical Sciences approved this study, and informed consent was obtained from all patients. Paired bone marrow DNA samples from 4 adult patients with B-ALL at diagnosis (more than 80% bone marrow involvement by leukemia) and in remission (less than 5% involvement) were conducted for WES. These four patients were not detected with any known cytogenetic and molecular abnormality through chromosome G-banding karyotype analysis and RT-PCR for lymphoid and myeloid related fusion genes or mutations at diagnosis, including MLL/AFX, MLL/AF6, MLL/AF1q, MLL/AF1p, MLL/AF10, MLL/AF9, MLL/ENL, MLL/ELL, dupMLL, NPM/ALK, MLL/AF17, MLL/AF4, SIL/TAL1, E2A/Pbx1, TEL/AML1, E2A/HLF, NPM/MLF1, AML1/MDS1/EVI1, TEL/ABL, TEL/PDGFR, DEK/CAN, CBFβ/MYH11, AML1/ETO, SET/CAN, BCR/ABL1, TLS/ERG, NPM/RARα, PML/RARα, PLZF/RARα, HOX11, EVI1, FLT3-ITD, c-kit, NPM1, C/EBPα and JAK2. In addition, genomic DNA samples isolated from 205 patients with ALL (B-ALL, *n* = 186; T-ALL, *n* = 19) were sequenced for *JAK1* using Sanger sequencing. Among them, 53 patients (B-ALL, *n* = 46; T-ALL, *n* = 7) were analyzed for all exons of *JAK1*, and 152 patients (B-ALL, *n* = 140; T-ALL, *n* = 12) were analyzed for exons 14 and 21 of *JAK1*.

### Exome sequencing and mutation analysis

As previously reported, exome sequencing was performed on 4 adult patients with B-ALL [40, 41]. In brief, an exome capturing sequencing library was produced from Agilent's SureSelect Human All Exon V5, followed by sequencing on the Illumina HiSeqTM2000 platform. Candidate variants were confirmed by Sanger sequencing. Mutation analysis for *JAK1* in a large cohort was performed by PCR amplification of specific exons and Sanger sequencing.

### Cell culture and cytokines

BaF3 murine pro-B cells was obtained from the China Infrastructure of Cell Line Resources, cultured in RPMI 1640 medium (Hyclone, Logan, Utah, USA) with 10% fetal bovine serum (FBS) (Gibco, California, USA) and 10 ng/ml mIL-3 (Peprotech, Rocky Hill, USA). NALM-6 human B-ALL cells (Biopike, Beijing, China) were cultured in RPMI 1640 medium with 10% FBS. HEK293V cells (Inovogen, Beijing, China) were cultured in DMEM medium (Hyclone, Logan, Utah, USA) with 10% FBS.

### Plasmid constructs, virus production and transfection

Human *JAK1* cDNA was obtained from pDONR223_JAK1 (Addgene, Cambridge, MA, USA), and subcloned into the lentiviral expression vector pLV-EGFP(2A)Puro (Inovogen, Beijing, China). The mutations resulting in A639G, S646P, P960S, and V658F substitutions were introduced into *JAK1* coding sequence by site-directed mutagenesis and confirmed by full-length DNA sequencing. Lentiviral supernatants were generated by co-transfection of lentiviral packaging plasmids and expression vector into HEK293V cells using CaCl_2_, harvested at 48 h and 72 h post-transfection. The supernatants were then concentrated and purified using Amicon^®^ Pro Purification System (Merck Millipore, Darmstadt, Germany). To estimate viral titers, HEK293V cells were incubated for 72 h with graded amounts of viral supernatant. BaF3 and NALM-6 cells were infected with the concentrated lentiviral supernatants (MOI = 10) and 8 μg/mL polybrene (Sigma-Aldrich, St Louis, MO, USA). The expression of GFP was analyzed by Accuri C6 (BD Biosciences, San Jose, CA, USA). Transduced BaF3 and NALM-6 cells were selected in growth media containing 1 μg/ml puromycin (Sigma-Aldrich, St Louis, MO, USA) for 7 days.

### Cell proliferation and apoptosis assays

Transduced BaF3 cells were washed three times with PBS, 1 × 10^5^ cells/well were seeded in 96-well plates, cultured in RPMI 1640 medium supplemented with 10% FBS in the absence of IL-3. Three days later, cell proliferation was assessed using a Cell Counting Kit 8 (CCK-8, Dojindo, Tokyo, Japan) assay as previously described [42]. The cell growth curves of transduced BaF3 were also determined using this assay. In brief, 2000 cells/well were seeded in 96-well plates and cultured in the absence of IL-3.10 μl CCK-8 was added to the wells at the indicated time points for 2 h, and the absorbance at 450 nm was measured by iMark (Bio-rad, Hercules, California, USA). Cell viability after withdrawal of IL-3 for 48 h was checked by trypan blue exclusion method.

### Cell cycle analysis

2 × 10^6^ IL-3-dependent BaF3 cells or autonomous BaF3 cells were cultured in the presence or absence of mIL-3, respectively. Cells were washed twice with PBS and fixed with 70% (v/v) ethanol at 4 °C overnight, then were washed twice and incubated in PBS that contains 100 μg/ml RNaseA and 50 μg/ml propidium iodide (Sigma-Aldrich, St Louis, MO, USA) for 30 min. Cell cycle parameters were determined by Accuri C6 (BD Biosciences, San Jose, CA, USA). All data were recorded and analyzed using Flowjo software (Treestar, Version 10.1).

### Western blotting

1 × 10^6^ HEK293V cells were transiently transfected with 5 μg of empty vector (vector), wild-type (WT) or *JAK1* mutant (A639G, S646P, P960S and V658F) plasmid using vigofect (Vigorous Biotechnology, Beijing, China). Cell lysate preparation, gel electrophoresis and transfer to polyvinylidene difluoride (PVDF) membranes were performed as previously described [33]. The phosphorylation of signaling proteins was investigated with the following phospho-specific antibodies: anti-phospho-JAK1 (Tyr1022/1023), anti-phospho-ERK (Thr202/Tyr204) and anti-phospho-AKT (Ser473) antibodies were purchased from Cell Signaling Technology (CST, Danvers, MA, USA). Anti-phospho-STAT3 (Tyr705) antibody was purchased from Abcam (Cambridge, UK). Blots were re-probed with anti-JAK1 (Abcam, Cambridge, UK), anti-STAT3 (Abcam, Cambridge, UK), anti-ERK1/2 (CST, Danvers, MA, USA), anti-AKT (CST, Danvers, MA, USA), and anti-GAPDH (Transgen biotech, Beijing, China) antibodies.

### Animal tumorigenesis in nude mice

The Institutional Review Board of the Affiliated Hospital of the Academy of Military Medical Sciences approved all mouse experiments. To test the oncogenic ability of *JAK1* S646P mutant *in vivo*, 2 × 10^6^ BaF3 cells transduced with vector, WT or *JAK1* S646P mutant were injected intravenously into nude mice aged 5 to 6 weeks. Blood counts were monitored weekly after 15 days post-inoculation. After sacrificing the mice, their livers and spleens were photographed and weighed. The bone marrow smears were stained with Wright and analyzed for the blasts infiltration. Single-cell suspensions were prepared from peripheral blood and bone marrow, and were analyzed for the percentage of GFP-positive cells by flow cytometry.

### Statistics

The experimental data are shown as the mean value with bars representing the standard deviation of the mean (s.d.). Data were analyzed using GraphPad Prism Version 6.0. Statistical significance was determined using student's *t*-test for comparison of two groups or ANOVA with multiple groups. The comparison of occurrence of *JAK1* mutations in B-ALL and T-ALL was performed using chi-square test. *P* values < 0.05 were considered statistically significant.

## SUPPLEMENTARY MATERIALS FIGURES AND TABLES






