# Supplementary material for: Identification of a novel functional JAK1 S646P mutation in acute lymphoblastic leukemia
Source: Oncotarget. 2017 Mar 29;8(21):34687–97. doi: 10.18632/oncotarget.16670 (PMC5471003; doi:10.18632/oncotarget.16670)
Supplement: Supplementary file 1 [file oncotarget-08-34687-s001.pdf]

Identification of a novel functional JAK1 S646P mutation in acute lymphoblastic leukemia

Supplementary Materials

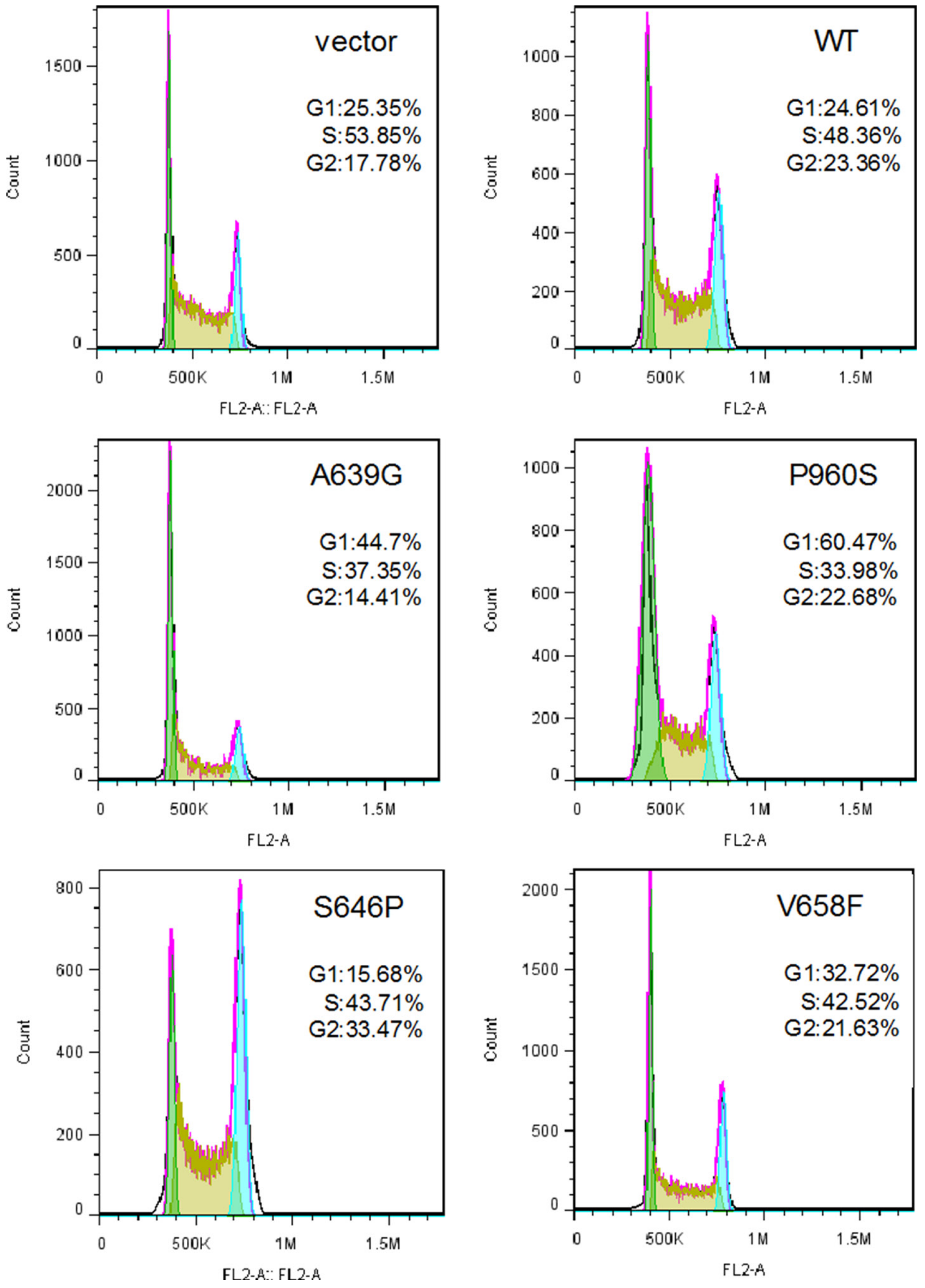

Supplementary Figure 1: Cell cycle histograms of transduced NALM-6 cells. Cell cycle histograms of transduced NALM-6cells were assayed by flow cytometry. Numbers indicate the percentage of cells in different phases of cell cycle.

Supplementary Table 1: Validated candidate genes selected from whole-exome sequencing data

Chromosome	Position	Gene	Normal/Variant	Amino acid site	Amino acid substitution
11	228474654	OBSCN	T/A	3337	L/H
	228529847		C/T	6733	A/V
19	1116897	SBNO2	C/T	602	G/D
	1112893		T/C	775	E/G
1	65316494	JAK1	A/G	583	L/P
	65312383		A/G	646	S/P
X	39913526	BCOR	T/C	1601	Y/C
11	9598724	WEE1	T/A	352	V/E
1	204495527	MDM4	G/A	40	A/T
X	54359928	WNK3	C/A	60	R/L
3	193854778	HES1	C/T	78	A/V
12	7947092	NANOG	A/T	152	R/W
3	45153637	CDCP1	A/G	198	L/P
17	33267588	CCT6B	A/G	313	L/P
19	2191137		T/C	131	F/L
	2191146	DOT1L	G/A	134	E/K
	2211209		T/C	488	L/P
X	70316698	FOXO4	C/A	107	A/E
16	89345527	ANKRD11	A/G	2475	S/P
4	40245187	RHOH	G/A	61	G/S
3	49722460	MST1	T/A	536	Q/L
8	22862925	RHOBTB2	T/C	100	V/A
1	214567111	PTPN14	C/A	286	E/*
16	3778156	CREBBP	G/A	2328	Q/*
3	10076890	FANCD2	A/T	137	K/N
3	172523578	ECT2	C/T	691	R/*
17	18140964	LLGL1	A/T	594	Q/L
X	128599865	SMARCA1	T/C	925	R/G
19	17953872	JAK3	T/A	177	Q/L

Supplementary Table 2: All SNVs from whole-exome sequencing data. See Supplementary_Table_2

Supplementary Table 3: Genetic characteristics of the 209 patients with ALL. See Supplementary_Table_3
