# Supplementary material for: Identification of a novel functional JAK1 S646P mutation in acute lymphoblastic leukemia
Source: Oncotarget. 2017 Mar 29;8(21):34687–97. doi: 10.18632/oncotarget.16670 (PMC5471003; doi:10.18632/oncotarget.16670)
Supplement: Supplementary file 2 [file oncotarget-08-34687-s002.docx]

Table S2 All SNVs from whole-exome sequencing data

| NO. | GENE | Base substitution | AA position | Ref | Alt | Significance |
| --- | --- | --- | --- | --- | --- | --- |
| 1 | JAK1 | GAT C[T/C]G GTG | 583 | L | P | Single AA Change |
|  |  | GTC [T/C]CC CAC | 646 | S | P | Single AA Change |
| 2 | CYR61 | GAC G[G/A]C CGA | 320 | G | D | Single AA Change |
|  |  | GCG C[T/A]C GCC | 9 | L | H | Single AA Change |
| 3 | NTNG1 | CTC C[T/A]G CAC | 472 | L | Q | Single AA Change |
| 4 | ETV3 | AAT [T/C]AT GAC | 87 | Y | H | Single AA Change |
| 5 | HMCN1 | ATA G[T/C]T TCT | 4927 | V | A | Single AA Change |
| 6 | LMOD1 | ATG G[A/G]G GAG | 30 | E | G | Single AA Change |
| 7 | OBSCN | CTG G[C/T]C GAC | 6733 | A | V | Single AA Change |
|  |  | CTG C[T/A]C ATC | 3337 | L | H | Single AA Change |
| 8 | SOCS5 | CCC C[C/T]T CCC | 279 | P | L | Single AA Change |
| 9 | ZNF514 | TTT CA[C/A] ACT | 366 | H | Q | Single AA Change |
| 10 | ARHGEF4 | GAG [T/C]GT GAG | 359 | C | R | Single AA Change |
| 11 | ZNF804A | TTC [C/T]GG GGC | 26 | R | W | Single AA Change |
| 12 | RPSA | CTT G[C/A]A GCA | 20 | A | E | Single AA Change |
| 13 | PCOLCE2 | ATG G[G/A]C CAA | 380 | G | D | Single AA Change |
| 14 | HES1 | AAG G[C/T]G GAC | 78 | A | V | Single AA Change |
| 15 | SPATA4 | TGG G[C/T]A CAG | 112 | A | V | Single AA Change |
| 16 | RASGRF2 | TCC A[A/G]A ATG | 1181 | K | R | Single AA Change |
| 17 | PCDHB1 | TGT G[G/A]C AAA | 97 | G | D | Single AA Change |
| 18 | SLC25A27 | CAT C[A/T]T GCA | 175 | H | L | Single AA Change |
| 19 | UNCX | GGG C[G/A]G CCG | 172 | R | Q | Single AA Change |
| 20 | TMEM184A | AGC T[T/C]C CTG | 135 | F | S | Single AA Change |
| 21 | C7orf46 | AGA C[C/A]T GAA | 261 | P | H | Single AA Change |
| 22 | TECPR1 | GGC G[T/C]G GTG | 781 | V | A | Single AA Change |
| 23 | CYP3A43 | TTA [G/T]GT CCA | 109 | G | C | Single AA Change |
| 24 | NOS3 | ATT TT[C/A] GGG | 1160 | F | L | Single AA Change |
| 25 | OXR1 | ACA A[G/T]C TTG | 748 | S | I | Single AA Change |
| 26 | PLEC | CGT [G/T]GC CTG | 4024 | G | C | Single AA Change |
| 27 | FAM120A | AAC AA[G/T] GGC | 129 | K | N | Single AA Change |
| 28 | SCAI | CGG [G/C]AT ATT | 419 | D | H | Single AA Change |
| 29 | MAPKAP1 | CTG C[A/G]T ATT | 233 | H | R | Single AA Change |
| 30 | NPDC1 | TCG [G/T]AT GAG | 355 | D | Y | Single AA Change |
| 31 | SPTBN2 | CGC A[C/T]C GTG | 352 | T | I | Single AA Change |
| 32 | CORO1B | GAG G[G/A]G CAC | 130 | G | E | Single AA Change |
| 33 | DSCAML1 | ACC T[A/G]C CAC | 1997 | Y | C | Single AA Change |
| 34 | ERC1 | CAA G[A/G]G GAA | 645 | E | G | Single AA Change |
| 35 | NANOG | AAG [A/T]GG TGG | 152 | R | W | Single AA Change |
| 36 | LTA4H | AAA G[T/C]T CCA | 196 | V | A | Single AA Change |
| 37 | CCDC92 | TTG [C/T]GG GAA | 234 | R | W | Single AA Change |
| 38 | MAPK1IP1L | CCA C[C/A]A GGA | 196 | P | Q | Single AA Change |
| 39 | PTPN21 | CCC G[T/A]G GCG | 613 | V | E | Single AA Change |
| 40 | AMN | GCC GA[G/T] CGC | 133 | E | D | Single AA Change |
| 41 | JAG2 | AAC C[G/A]C GCG | 1224 | R | H | Single AA Change |
| 42 | GNB5 | CCC [A/C]GT GGA | 292 | S | R | Single AA Change |
| 43 | B3GNT9 | GCC [T/C]AC GCG | 286 | Y | H | Single AA Change |
| 44 | LLGL1 | GTG C[A/T]G TGC | 594 | Q | L | Single AA Change |
| 45 | MMP28 | GCC [T/C]TC CAG | 158 | F | L | Single AA Change |
| 46 | SLC14A2 | TTG [A/G]GA GCC | 746 | R | G | Single AA Change |
| 47 | SBNO2 | TTC G[A/G]G TCG | 775 | E | G | Single AA Change |
|  |  | ACG G[G/A]C GAG | 602 | G | D | Single AA Change |
| 48 | APC2 | TGC [G/A]CC TCC | 1275 | A | T | Single AA Change |
| 49 | ARHGEF18 | CAG [A/T]GC CTG | 696 | S | C | Single AA Change |
| 50 | PRKCSH | AAC [C/A]GC TCC | 477 | R | S | Single AA Change |
| 51 | LPHN1 | AAC [G/A]GG CTG | 276 | G | R | Single AA Change |
| 52 | KLK14 | ACC T[C/A]C TGC | 158 | S | Y | Single AA Change |
| 53 | ITIH6 | CTT A[C/A]C CTC | 1198 | T | N | Single AA Change |
| 54 | FAM3A | GAC A[G/T]C TGG | 189 | S | I | Single AA Change |
| 55 | KDM5D | GAT [A/G]GC CAG | 580 | S | G | Single AA Change |
| 56 | CCDC138 | GCA TT[T/A] GAT | 513 | F | L | Single AA Change |
| 57 | C3orf77 | TCT G[C/T]C CTG | 1672 | A | V | Single AA Change |
| 58 | EPB41L4A | CTG [C/T]CC TGT | 129 | P | S | Single AA Change |
| 59 | PAX6 | TCC A[C/T]C CGG | 25 | T | I | Single AA Change |
| 60 | NME3 | AAG T[A/T]T ATG | 84 | Y | F | Single AA Change |
| 61 | RAB5C | CTC G[C/T]G GGT | 165 | A | V | Single AA Change |
| 62 | ZNF493 | CTT A[C/A]T AAA | 477 | T | N | Single AA Change |
| 63 | TCTEX1D1 | TCT [G/T]AG GTT | 112 | E | * | Nonsense |
| 64 | VCPIP1 | GCT T[C/A]G GGG | 33 | S | * | Nonsense |
| 65 | FOLH1 | CAG T[G/A]G AAA | 100 | W | * | Nonsense |
| 66 | C14orf39 | AAT [C/T]AG TGG | 365 | Q | * | Nonsense |
| 67 | CREBBP | CAG [C/T]AA CAG | 2328 | Q | * | Nonsense |
| 68 | CASKIN2 | GCT [G/T]AG GGG | 812 | E | * | Nonsense |
| 69 | MYO9B | GAG [C/T]AG ATG | 1884 | Q | * | Nonsense |
| 70 | SIGLEC1 | GCA [C/T]AA AAG | 459 | Q | * | Nonsense |
| 71 | NIPSNAP1 | TGG [C/T]GA TTC | 127 | R | * | Nonsense |
| 72 | ZNF644 | CAG C[A/T]T TTA | 938 | H | L | Single AA Change |
| 73 | GON4L | AGG C[G/A]C ATG | 1505 | R | H | Single AA Change |
| 74 | TRMT1L | GGA [A/G]AG AGA | 609 | K | E | Single AA Change |
| 75 | GAL3ST2 | AT[G/A] ATG | 1 | M | I | Single AA Change |
| 76 | CAND2 | CAG [C/A]TG GAA | 152 | L | M | Single AA Change |
| 77 | PFN2 | TAC [T/A]TG AGA | 135 | L | M | Single AA Change |
| 78 | LIN28B | TCT C[A/G]G GGA | 177 | Q | R | Single AA Change |
| 79 | GPR126 | TCC AA[A/T] TCT | 1176 | K | N | Single AA Change |
| 80 | LFNG | GCC [C/A]TG CTG | 210 | L | M | Single AA Change |
| 81 | KIAA0415 | ATC [C/A]TG GTT | 610 | L | M | Single AA Change |
| 82 | TNRC18 | GCT G[G/T]C CTA | 475 | G | V | Single AA Change |
| 83 | GCK | TAC [A/G]TG GGC | 299 | M | V | Single AA Change |
| 84 | FAM82B | AAT G[C/T]A TAT | 187 | A | V | Single AA Change |
| 85 | SLC35G1 | CTT T[T/A]C TCA | 82 | F | Y | Single AA Change |
| 86 | POU2F3 | AAT [G/T]AT CGA | 37 | D | Y | Single AA Change |
| 87 | ARID2 | CAT C[T/A]G ATG | 354 | L | Q | Single AA Change |
| 88 | C12orf63 | GTA TT[C/A] CTT | 596 | F | L | Single AA Change |
| 89 | SCYL2 | GTC AT[A/G] AAA | 595 | I | M | Single AA Change |
| 90 | ZMYM2 | AAA A[G/T]T CCA | 1314 | S | I | Single AA Change |
| 91 | NAA30 | GCA C[C/A]T CCG | 17 | P | H | Single AA Change |
| 92 | ATXN3 | GAA G[C/T]C TAC | 287 | A | V | Single AA Change |
| 93 | HERC1 | GCA C[T/C]T TCT | 1201 | L | P | Single AA Change |
| 94 | RNF40 | AGC [A/G]GC AGT | 173 | S | G | Single AA Change |
| 95 | FAM65C | GGG [G/T]TC GTG | 22 | V | F | Single AA Change |
| 96 | PPP1R3F | TGG [C/T]CC CAG | 376 | P | S | Single AA Change |
| 97 | KIF5C | ATT G[T/C]C AAA | 71 | V | A | Single AA Change |
| 98 | MYO1B | AAA [A/T]GG ATT | 916 | R | W | Single AA Change |
| 99 | NEU4 | GTC TT[C/G] CTC | 112 | F | L | Single AA Change |
| 100 | SDHA | GTG [C/T]CC GGC | 433 | P | S | Single AA Change |
| 101 | SLCO4C1 | AAC TT[G/T] ATA | 497 | L | F | Single AA Change |
| 102 | DDX46 | AGA G[T/C]A GAA | 167 | V | A | Single AA Change |
| 103 | B4GALT7 | GAA [C/T]GC TTC | 104 | R | C | Single AA Change |
| 104 | PRRT1 | ATT [G/T]CC ATC | 242 | A | S | Single AA Change |
| 105 | PKD1L1 | GCT T[C/T]G AAG | 1175 | S | L | Single AA Change |
| 106 | NRCAM | AAG G[A/T]C AAC | 584 | D | V | Single AA Change |
| 107 | IMPDH1 | CTG G[T/C]G GGC | 240 | V | A | Single AA Change |
| 108 | ZNF212 | CTG [G/A]AG AAC | 112 | E | K | Single AA Change |
| 109 | INTS9 | CTG G[A/T]C ATG | 31 | D | V | Single AA Change |
| 110 | PYCRL | GGC C[T/G]C AGT | 188 | L | R | Single AA Change |
| 111 | SCRIB | AGC [G/T]TC CTC | 339 | V | F | Single AA Change |
| 112 | CDHR1 | GAG G[G/A]A GAC | 64 | G | E | Single AA Change |
| 113 | PPAPDC1A | GAA G[C/A]C TTC | 83 | A | D | Single AA Change |
| 114 | PSMD13 | ACC [C/T]TC TAT | 239 | L | F | Single AA Change |
| 115 | ZDHHC5 | TCA G[A/G]T GGG | 275 | D | G | Single AA Change |
| 116 | POU6F1 | GAA G[G/A]C CAG | 219 | G | D | Single AA Change |
| 117 | ERCC5 | ACC C[C/A]T GGC | 1350 | P | H | Single AA Change |
| 118 | ASB2 | ATG [A/G]AG TGC | 487 | K | E | Single AA Change |
| 119 | PLCB2 | CAT G[A/G]G TTC | 659 | E | G | Single AA Change |
| 120 | C15orf63 | CGG G[A/G]G AAA | 82 | E | G | Single AA Change |
| 121 | IGDCC3 | TAT [C/G]AG TGT | 399 | Q | E | Single AA Change |
| 122 | FUK | AGC G[T/A]C CTG | 415 | V | D | Single AA Change |
| 123 | GALNS | AAT TT[G/T] GAC | 59 | L | F | Single AA Change |
| 124 | ANKRD11 | GGC [T/C]CC TAC | 2475 | S | P | Single AA Change |
| 125 | DBNDD1 | GAC G[A/T]C GAG | 217 | D | V | Single AA Change |
| 126 | RASD1 | ATC [T/A]CG GCC | 177 | S | T | Single AA Change |
| 127 | CCT6B | GCT C[T/C]T CGC | 313 | L | P | Single AA Change |
| 128 | BTBD2 | GAC [G/A]CC ATC | 252 | A | T | Single AA Change |
| 129 | DOT1L | CCC [T/C]TC TCC | 131 | F | L | Single AA Change |
|  | DOT1L | CCC [G/A]AG GTG | 134 | E | K | Single AA Change |
|  | DOT1L | CTT C[T/C]A GAG | 488 | L | P | Single AA Change |
| 130 | JAK3 | GAG C[A/T]G GCC | 177 | Q | L | Single AA Change |
| 131 | CILP2 | GAG C[T/C]G GAA | 575 | L | P | Single AA Change |
| 132 | ZNF14 | ACT G[G/T]A GTG | 165 | G | V | Single AA Change |
| 133 | TDRD12 | GGT T[G/T]C TTC | 14 | C | F | Single AA Change |
| 134 | EXOC3L2 | GAG G[G/A]A GCC | 349 | G | E | Single AA Change |
| 135 | PPFIA3 | GAA GA[G/T] AAG | 408 | E | D | Single AA Change |
| 136 | UCKL1 | CGC [C/T]GG GAC | 240 | R | W | Single AA Change |
| 137 | FOXO4 | TAT G[C/A]A GAA | 107 | A | E | Single AA Change |
| 138 | EFNA3 | TTC C[A/G]G CGC | 127 | Q | R | Single AA Change |
| 139 | EML6 | ACC [C/A]GC GAG | 50 | R | S | Single AA Change |
| 140 | PAWR | AAC C[T/C]A AGA | 287 | L | P | Single AA Change |
| 141 | CYB561 | TGG [A/G]AG CGG | 292 | K | E | Single AA Change |
| 142 | C3 | AAC C[C/A]C ATG | 702 | P | H | Single AA Change |
| 143 | FAM123C | GCC T[C/A]G CCC | 297 | S | * | Nonsense |
| 144 | LIMCH1 | CGA [C/T]GA TCC | 753 | R | * | Nonsense |
| 145 | BTAF1 | GTG [G/T]AG CAT | 1725 | E | * | Nonsense |
| 146 | AHNAK2 | TCG [G/T]GA CAG | 333 | G | * | Nonsense |
| 147 | PRSS8 | TGG [G/T]GA GAT | 259 | G | * | Nonsense |
| 148 | YIF1B | TAC T[G/A]G CTC | 307 | W | * | Nonsense |
| 149 | C22orf15 | TCT TG[C/A] AGG | 19 | C | * | Nonsense |
| 150 | RP4-788L13.1.1 | CCC [G/A]AA AAC | 745 | E | K | Single AA Change |
| 151 | NES | GGG AT[G/A] GAG | 1523 | M | I | Single AA Change |
| 152 | GPR113 | GGC [T/C]AC AAG | 16 | Y | H | Single AA Change |
| 153 | XIRP1 | GGC A[G/C]C AGT | 867 | S | T | Single AA Change |
| 154 | RAB33B | GCA A[G/A]C TTT | 12 | S | N | Single AA Change |
| 155 | HTR4 | AGT [G/T]AC ACT | 427 | D | Y | Single AA Change |
| 156 | DENND3 | GGG CA[G/T] GGA | 1222 | Q | H | Single AA Change |
| 157 | STK32C | GAT G[C/T]T GCG | 473 | A | V | Single AA Change |
| 158 | TAC3 | TCT [C/A]TG GAG | 57 | L | M | Single AA Change |
| 159 | BTBD1 | CTG [A/G]GC GAT | 69 | S | G | Single AA Change |
| 160 | PIGQ | GTC [T/G]CT GCA | 281 | S | A | Single AA Change |
| 161 | P2RX5 | AGC A[G/T]C AGT | 403 | S | I | Single AA Change |
| 162 | PIK3R5 | ACG C[C/T]C TCA | 580 | P | L | Single AA Change |
| 163 | HS3ST3A1 | TTC [C/G]TG CGC | 172 | L | V | Single AA Change |
| 164 | MED1 | GGC A[G/A]C AAA | 1094 | S | N | Single AA Change |
| 165 | MKS1 | AGC [C/A]TC CCG | 546 | L | I | Single AA Change |
| 166 | SMCHD1 | ACG A[C/A]A GAT | 1980 | T | K | Single AA Change |
| 167 | TSSK6 | GCC [G/A]TG CAG | 101 | V | M | Single AA Change |
| 168 | CEBPA | GCG [A/T]GT GGC | 266 | S | C | Single AA Change |
| 169 | WDR62 | TGG AT[C/G] AAC | 291 | I | M | Single AA Change |
| 170 | ARHGEF1 | GAG G[G/A]C CTG | 876 | G | D | Single AA Change |
| 171 | LONRF3 | GGG [C/T]GG GCC | 186 | R | W | Single AA Change |
| 172 | HCFC1 | TCC T[C/T]C ACA | 1478 | S | F | Single AA Change |
| 173 | RPS8 | GTT C[G/A]T ACC | 92 | R | H | Single AA Change |
| 174 | NRAS | GAG [T/G]AC AGT | 64 | Y | D | Single AA Change |
| 175 | NAV1 | AAT G[C/A]T AAT | 1108 | A | D | Single AA Change |
| 176 | GLI2 | TGC [C/T]GC TGG | 473 | R | C | Single AA Change |
| 177 | SCN7A | CAA G[T/A]T GCA | 1430 | V | D | Single AA Change |
| 178 | CDCP1 | CAC C[T/C]C CCA | 198 | L | P | Single AA Change |
| 179 | MST1 | CGG C[A/T]G TGC | 536 | Q | L | Single AA Change |
| 180 | COL6A5 | CAG C[T/A]T GGG | 2139 | L | H | Single AA Change |
| 181 | KIAA0226 | AAG C[A/T]G AAG | 537 | Q | L | Single AA Change |
| 182 | RHOH | GCC [G/A]GC AAT | 61 | G | S | Single AA Change |
| 183 | PDLIM5 | GAT G[G/A]A ATA | 55 | G | E | Single AA Change |
| 184 | RREB1 | CAG [C/A]AC CTG | 813 | H | N | Single AA Change |
| 185 | RNF144B | TGC [C/T]GG GTT | 197 | R | W | Single AA Change |
| 186 | CDK13 | AGT C[C/T]A GAG | 665 | P | L | Single AA Change |
| 187 | FAM167A | ATG [C/G]GG CTG | 131 | R | G | Single AA Change |
| 188 | RHOBTB2 | AGC G[T/C]C TCT | 100 | V | A | Single AA Change |
| 189 | WDR67 | ACG C[C/A]T ACT | 654 | P | H | Single AA Change |
| 190 | ACTL7A | TTT [G/C]AA GCC | 189 | E | Q | Single AA Change |
| 191 | PRKG1 | ACA G[C/A]A AAC | 318 | A | E | Single AA Change |
| 192 | NRAP | GAG AA[G/T] ACA | 553 | K | N | Single AA Change |
| 193 | CASP4 | GTA CA[G/T] CAA | 347 | Q | H | Single AA Change |
| 194 | RAPGEF3 | TCG [G/A]CC ATC | 765 | A | T | Single AA Change |
| 195 | TAOK3 | CAG T[T/C]T CAG | 723 | F | S | Single AA Change |
| 196 | GCNT3 | AAT G[G/A]G AGG | 248 | G | E | Single AA Change |
| 197 | ZKSCAN2 | TGT C[C/T]G CAA | 110 | P | L | Single AA Change |
| 198 | GUCY2D | TAC [C/T]GC ATC | 1017 | R | C | Single AA Change |
| 199 | ACACA | CAG [T/C]AC CTG | 1031 | Y | H | Single AA Change |
| 200 | USP32 | ATG [G/T]GT GCC | 2 | G | C | Single AA Change |
| 201 | MBD1 | CTG [C/T]CT CCA | 341 | P | S | Single AA Change |
| 202 | DCC | CGC [A/G]GG GGT | 668 | R | G | Single AA Change |
| 203 | POLRMT | CAC T[C/T]G CTG | 1126 | S | L | Single AA Change |
| 204 | TM6SF2 | ACC T[C/T]G GTT | 75 | S | L | Single AA Change |
| 205 | ZNF541 | CAC [G/A]AG GCT | 1148 | E | K | Single AA Change |
| 206 | CXorf26 | AAC [C/T]GG GAA | 151 | R | W | Single AA Change |
| 207 | SMARCA1 | GCA [A/G]GA ATT | 925 | R | G | Single AA Change |
| 208 | NOTO | CAG CA[A/T] AAG | 215 | Q | H | Single AA Change |
| 209 | RARS | TCG [G/A]GT GAA | 457 | G | S | Single AA Change |
| 210 | KIF6 | AAG C[A/T]C CGT | 291 | H | L | Single AA Change |
| 211 | PMS2 | GAG [T/C]AC GGT | 255 | Y | H | Single AA Change |
| 212 | GRHPR | GCG [G/A]GG GCC | 51 | G | R | Single AA Change |
| 213 | C11orf82 | GAG [T/A]GC CAT | 856 | C | S | Single AA Change |
| 214 | PIEZO1 | TTC [C/T]CT GCC | 653 | P | S | Single AA Change |
| 215 | KCNH4 | GTG [C/G]TC CGA | 604 | L | V | Single AA Change |
| 216 | KIF18B | AAA TG[G/T] GGT | 54 | W | C | Single AA Change |
| 217 | APOH | TGC [C/T]AT GAT | 235 | H | Y | Single AA Change |
| 218 | ZNF674 | TCT G[C/T]C CAG | 28 | A | V | Single AA Change |
| 219 | SPAG17 | AAG [A/T]AG TAC | 533 | K | * | Nonsense |
| 220 | BNIPL | GAT [C/T]AA GTC | 337 | Q | * | Nonsense |
| 221 | PLA2G12A | GAA [G/T]AA TTC | 127 | E | * | Nonsense |
| 222 | RNMT | GAA TA[T/A] ATT | 251 | Y | * | Nonsense |
| 223 | LRRC8D | CAA [T/G]TG TTT | 771 | L | V | Single AA Change |
| 224 | MYNN | GTA [G/A]AA CAA | 224 | E | K | Single AA Change |
| 225 | STK31 | TTA [A/T]GT GAG | 260 | S | C | Single AA Change |
| 226 | ZNF479 | AAT GA[G/T] ATG | 81 | E | D | Single AA Change |
| 227 | MRPL15 | CCT G[C/T]C AAA | 227 | A | V | Single AA Change |
| 228 | RTN3 | GTG A[C/A]A ACA | 630 | T | K | Single AA Change |
| 229 | CDON | CCA [C/T]CC GTG | 690 | P | S | Single AA Change |
| 230 | ESD | AAG C[G/A]G ATT | 217 | R | Q | Single AA Change |
| 231 | RASIP1 | ATG [G/A]CC AAC | 651 | A | T | Single AA Change |
| 232 | DBT | ATA A[A/T]A GGC | 257 | K | I | Single AA Change |
| 233 | ATF6 | CCA C[C/T]T CCT | 465 | P | L | Single AA Change |
| 234 | MDM4 | GCA [G/A]CA GGT | 40 | A | T | Single AA Change |
| 235 | GCFC2 | CGC [A/G]AT ATT | 656 | N | D | Single AA Change |
| 236 | SCN1A | GCA T[T/C]G AAG | 224 | L | S | Single AA Change |
| 237 | XIRP2 | GGT [G/T]AT TTG | 1840 | D | Y | Single AA Change |
| 238 | ALS2CR11 | ACT G[T/C]T GGG | 300 | V | A | Single AA Change |
| 239 | FANCD2 | ATC AA[A/T] CTG | 137 | K | N | Single AA Change |
| 240 | RARB | AAG [G/A]GC TTT | 110 | G | S | Single AA Change |
| 241 | MON1A | CTG C[G/A]T CAC | 543 | R | H | Single AA Change |
| 242 | PHF7 | AGG G[G/A]C CAG | 72 | G | D | Single AA Change |
| 243 | WNT5A | GCC C[G/A]C GAG | 200 | R | H | Single AA Change |
| 244 | ZNF717 | CTT C[T/C]C ACT | 382 | L | P | Single AA Change |
| 245 | KLF15 | GAG AA[G/T] AAG | 388 | K | N | Single AA Change |
| 246 | CPNE4 | GGC TG[C/G] CAA | 322 | C | W | Single AA Change |
| 247 | KIT | TTT [G/C]GA TCA | 297 | G | R | Single AA Change |
| 248 | DNAH5 | TGG C[T/C]T AAT | 1789 | L | P | Single AA Change |
| 249 | MCCC2 | ACT G[T/C]G AAA | 150 | V | A | Single AA Change |
| 250 | AP3B1 | GTT C[G/C]A AAT | 649 | R | P | Single AA Change |
| 251 | PCDHB4 | GGC [G/A]AG GTG | 622 | E | K | Single AA Change |
| 252 | ZFP2 | AAG CA[C/G] CAG | 120 | H | Q | Single AA Change |
| 253 | AMD1 | ACT T[A/G]T TGG | 239 | Y | C | Single AA Change |
| 254 | VIPR2 | TTG C[A/T]C TGC | 332 | H | L | Single AA Change |
| 255 | C8orf86 | ATG C[A/T]A ATC | 75 | Q | L | Single AA Change |
| 256 | ARFGEF1 | CCT C[G/T]C ATA | 94 | R | L | Single AA Change |
| 257 | RNH1 | AGC C[T/C]G GAC | 8 | L | P | Single AA Change |
| 258 | WEE1 | GCA G[T/A]G CTT | 352 | V | E | Single AA Change |
| 259 | OR10AG1 | ATG T[A/G]T TTT | 51 | Y | C | Single AA Change |
| 260 | PCNXL3 | GTG A[T/C]C GCG | 854 | I | T | Single AA Change |
| 261 | INTS4 | GAT G[T/A]T GAT | 847 | V | D | Single AA Change |
| 262 | TREH | GTG [C/T]CT GGC | 165 | P | S | Single AA Change |
| 263 | DHH | ACT G[G/T]C ATG | 379 | G | V | Single AA Change |
| 264 | RNFT2 | GGC G[C/T]C ACG | 436 | A | V | Single AA Change |
| 265 | DNAJC3 | CAT G[C/T]T GCC | 62 | A | V | Single AA Change |
| 266 | MNAT1 | CCA C[T/C]C AGA | 53 | L | P | Single AA Change |
| 267 | HIF1A | AGC [C/T]TC TTT | 410 | L | F | Single AA Change |
| 268 | CDAN1 | CCA AA[C/G] CTC | 171 | N | K | Single AA Change |
| 269 | ZNF280D | ATA AA[A/C] CCT | 667 | K | N | Single AA Change |
| 270 | ADAMTS7 | CCT [G/T]GG GTG | 801 | G | W | Single AA Change |
| 271 | ABCA3 | CAG [G/A]CC AAG | 1127 | A | T | Single AA Change |
| 272 | XYLT1 | AAG T[C/T]G GAG | 947 | S | L | Single AA Change |
| 273 | ERN2 | CTG G[C/T]C CAC | 674 | A | V | Single AA Change |
| 274 | GGT6 | TCC C[A/G]C GGC | 100 | H | R | Single AA Change |
| 275 | HNF1B | AGC [C/T]AC ATG | 494 | H | Y | Single AA Change |
| 276 | BAHCC1 | GTG C[C/A]T TCT | 396 | P | H | Single AA Change |
| 277 | OSBPL1A | ATC [A/C]CC TTG | 693 | T | P | Single AA Change |
| 278 | DSC1 | GTT C[T/C]G AAA | 593 | L | P | Single AA Change |
| 279 | RAX2 | GCG T[T/C]C GAG | 92 | F | S | Single AA Change |
| 280 | ICAM5 | GAC C[T/A]G CAG | 38 | L | Q | Single AA Change |
| 281 | SPTBN4 | AAC [C/T]GG TCG | 2440 | R | W | Single AA Change |
| 282 | TMC2 | TAT G[G/T]A GTT | 261 | G | V | Single AA Change |
| 283 | CBR1 | AAG G[G/A]C ATC | 16 | G | D | Single AA Change |
| 284 | CSF2RB | CAC C[T/C]C ATG | 202 | L | P | Single AA Change |
| 285 | ARSE | CTG CA[G/T] CTG | 583 | Q | H | Single AA Change |
| 286 | TXLNG | ATG G[A/G]A GAA | 41 | E | G | Single AA Change |
|  |  | AGA AG[C/A] AAG | 206 | S | R | Single AA Change |
| 287 | PHKA2 | CTG G[G/A]C ATG | 58 | G | D | Single AA Change |
| 288 | BCOR | TTC T[A/G]T GGC | 1601 | Y | C | Single AA Change |
| 289 | WNK3 | TTC C[G/T]A AAG | 60 | R | L | Single AA Change |
| 290 | H2BFM | CTG [G/A]CC CAT | 207 | A | T | Single AA Change |
| 291 | DDX26B | AAA [A/C]CT GGT | 280 | T | P | Single AA Change |
| 292 | ZIC3 | GTC [A/G]CC ATG | 283 | T | A | Single AA Change |
| 293 | SRPK3 | AAG A[T/C]C GGC | 138 | I | T | Single AA Change |
| 294 | WDR19 | GTG T[A/G]C ACT | 582 | Y | C | Single AA Change |
| 295 | C6orf70 | GAG G[A/G]T TTG | 242 | D | G | Single AA Change |
| 296 | RFC2 | GGC C[G/T]A CAT | 134 | R | L | Single AA Change |
| 297 | TCEB1 | TTA [A/G]CA TCA | 38 | T | A | Single AA Change |
| 298 | AMPD3 | CCT G[A/G]C AGG | 351 | D | G | Single AA Change |
| 299 | EZH1 | AAA C[G/T]C AAG | 324 | R | L | Single AA Change |
| 300 | PTPN14 | ATC [G/T]AA AAT | 286 | E | * | Nonsense |
| 301 | ECT2 | ACC [C/T]GA CCC | 691 | R | * | Nonsense |
| 302 | NPM2 | TTC [A/T]GA CCC | 33 | R | * | Nonsense |
| 303 | KIAA2026 | GCA [C/T]AA CCT | 1677 | Q | * | Nonsense |
| 304 | ZAR1L | CAG [G/T]AG GAG | 179 | E | * | Nonsense |
| 305 | ITGAD | CAC [A/T]GA GTC | 112 | R | * | Nonsense |
| 306 | SDK2 | CCT T[G/A]G GAA | 2187 | W | * | Nonsense |
| 307 | NOTCH2 | TAT G[C/T]G | 2471 | A | V | Single AA Change |
| 308 | B3GALT2 | CAC [C/T]GT AAA | 419 | R | C | Single AA Change |
| 309 | ALMS1 | TTC AG[T/A] CCA | 2104 | S | R | Single AA Change |
| 310 | LY75-CD302 | CTA G[G/C]C ATG | 1733 | G | A | Single AA Change |
| 311 | TOP2B | GCT T[C/A]T GTT | 1473 | S | Y | Single AA Change |
| 312 | SENP5 | GAA [A/G]TA ACA | 548 | I | V | Single AA Change |
| 313 | ADAMTS16 | ATG G[T/C]A AGA | 568 | V | A | Single AA Change |
| 314 | PTCD2 | AAC [A/G]AA AAT | 123 | K | E | Single AA Change |
| 315 | SHROOM1 | TTC C[T/C]A GAA | 391 | L | P | Single AA Change |
| 316 | NUP153 | GCT A[A/G]G CAA | 294 | K | R | Single AA Change |
| 317 | STXBP5 | ATA [A/T]TG ACT | 969 | M | L | Single AA Change |
| 318 | WNT16 | GGC T[A/G]C GAG | 111 | Y | C | Single AA Change |
| 319 | EPPK1 | CTA T[T/C]T CTT | 2414 | F | S | Single AA Change |
| 320 | PTCH1 | GTC C[G/T]G TGT | 1419 | R | L | Single AA Change |
| 321 | TSC1 | CAC [A/G]GA CAA | 434 | R | G | Single AA Change |
| 322 | UEVLD | GGA [A/G]AA CAT | 101 | K | E | Single AA Change |
| 323 | EML3 | CTC [A/G]GG CCC | 141 | R | G | Single AA Change |
| 324 | MLL | CCC C[C/T]G TCT | 773 | P | L | Single AA Change |
| 325 | NCKAP5L | CCC [A/G]GC ATG | 1194 | S | G | Single AA Change |
| 326 | SMG1 | ACT [C/A]TT CTT | 360 | L | I | Single AA Change |
| 327 | SPNS1 | GTG C[C/A]C GTG | 567 | P | H | Single AA Change |
| 328 | ADAT1 | GCG [G/A]TG CAG | 380 | V | M | Single AA Change |
| 329 | ZBTB4 | GAC G[G/T]G GCA | 157 | G | V | Single AA Change |
| 330 | KRT14 | AGC C[G/A]C CAA | 446 | R | H | Single AA Change |
| 331 | RANBP3 | CCT [C/T]CC GCT | 79 | P | S | Single AA Change |
| 332 | ATP13A1 | TCC C[G/A]G CTG | 380 | R | Q | Single AA Change |
| 333 | ZNF83 | CAT G[C/T]C GGT | 368 | A | V | Single AA Change |
| 334 | FAM19A5 | GGC [A/T]GC CGG | 9 | S | C | Single AA Change |
| 335 | KDM6A | TCA C[G/T]A AAG | 1358 | R | L | Single AA Change |
| 336 | NONO | TTC C[C/A]T GAT | 375 | P | H | Single AA Change |
| 337 | XIAP | TTA C[A/T]G AAA | 432 | Q | L | Single AA Change |
