# Supplementary material for: Identification of a novel functional JAK1 S646P mutation in acute lymphoblastic leukemia
Source: Oncotarget. 2017 Mar 29;8(21):34687–97. doi: 10.18632/oncotarget.16670 (PMC5471003; doi:10.18632/oncotarget.16670)
Supplement: Supplementary file 3 [file oncotarget-08-34687-s003.docx]

Table S3. Genetic characteristics of the 209 patients with ALL

| NO. | Gender | Age | Lineage | Genetic abnormalities |
| --- | --- | --- | --- | --- |
| 1 | M | 22 | B-ALL | - |
| 2 | M | 15 | B-ALL | - |
| 3 | M | 22 | B-ALL | - |
| 4 | F | 40 | B-ALL | - |
| 5 | F | 23 | B-ALL | - |
| 6 | M | 22 | B-ALL | TEL-AML1 |
| 7 | F | 23 | B-ALL | - |
| 8 | M | 24 | B-ALL | - |
| 9 | M | 27 | B-ALL | - |
| 10 | F | 54 | B-ALL | - |
| 11 | F | 40 | B-ALL | - |
| 12 | F | 42 | B-ALL | BCR-ABL |
| 13 | F | 56 | B-ALL | BCR-ABL |
| 14 | F | 48 | B-ALL | BCR-ABL |
| 15 | F | 40 | B-ALL | BCR-ABL |
| 16 | F | 30 | B-ALL | - |
| 17 | F | 23 | B-ALL | - |
| 18 | M | 22 | B-ALL | - |
| 19 | M | 48 | B-ALL | - |
| 20 | M | 27 | B-ALL | - |
| 21 | M | 56 | B-ALL | BCR-ABL |
| 22 | F | 60 | B-ALL | - |
| 23 | M | 25 | B-ALL | ELA2 |
| 24 | M | 56 | B-ALL | - |
| 25 | M | 22 | B-ALL | TEL-AML1 |
| 26 | F | 36 | B-ALL | BCR-ABL |
| 27 | F | 41 | B-ALL | BCR-ABL |
| 28 | F | 32 | B-ALL | - |
| 29 | M | 25 | B-ALL | - |
| 30 | F | 47 | B-ALL | - |
| 31 | F | 17 | B-ALL | - |
| 32 | M | 51 | B-ALL | BCR-ABL |
| 33 | F | 45 | B-ALL | BCR-ABL |
| 34 | F | 57 | B-ALL | - |
| 35 | F | 43 | B-ALL | BCR-ABL |
| 36 | F | 23 | B-ALL | - |
| 37 | M | 54 | B-ALL | - |
| 38 | F | 50 | B-ALL | - |
| 39 | F | 43 | B-ALL | - |
| 40 | F | 40 | B-ALL | - |
| 41 | M | 44 | B-ALL | - |
| 42 | M | 7 | B-ALL | - |
| 43 | F | 57 | B-ALL | - |
| 44 | M | 8 | B-ALL | - |
| 45 | M | 76 | B-ALL | - |
| 46 | F | 4 | B-ALL | - |
| 47 | F | 36 | B-ALL | E2A-PBX1 |
| 48 | F | 12 | B-ALL | - |
| 49 | M | 29 | B-ALL | BCR-ABL |
| 50 | F | 13 | B-ALL | MLL-ENL |
| 51 | F | 41 | B-ALL | EVI1, KIT |
| 52 | M | 27 | B-ALL | BCR-ABL |
| 53 | M | 22 | B-ALL | BCR-ABL |
| 54 | M | 42 | B-ALL | BCR-ABL |
| 55 | M | 30 | B-ALL | - |
| 56 | F | 50 | B-ALL | - |
| 57 | M | 17 | B-ALL | - |
| 58 | M | 21 | B-ALL | BCR-ABL |
| 59 | F | 58 | B-ALL | EVI1, BCR-ABL |
| 60 | F | 48 | B-ALL | HOX11, EVI1,BCR-ABL |
| 61 | M | 41 | B-ALL | HOX11, EVI1 |
| 62 | F | 33 | B-ALL | BCR-ABL |
| 63 | F | 22 | B-ALL | - |
| 64 | M | 19 | B-ALL | - |
| 65 | F | 29 | B-ALL | - |
| 66 | F | 46 | B-ALL | HOX11, EVI1 |
| 67 | M | 13 | B-ALL | - |
| 68 | F | 32 | B-ALL | - |
| 69 | F | 15 | B-ALL | HOX11, MLL-AF10 |
| 70 | M | 48 | B-ALL | HOX11, EVI1, MLL-ENL |
| 71 | M | 20 | B-ALL | BCR-ABL, HOX11, EVI1 |
| 72 | M | 20 | B-ALL | HOX11, EVI1, MLL-AF6 |
| 73 | M | 19 | B-ALL | HOX11, EVI1 |
| 74 | F | 24 | B-ALL | HOX11 |
| 75 | M | 42 | B-ALL | BCR-ABL |
| 76 | F | 39 | B-ALL | - |
| 77 | M | 25 | B-ALL | BCR-ABL |
| 78 | M | 21 | B-ALL | - |
| 79 | M | 17 | B-ALL | - |
| 80 | M | 40 | B-ALL | - |
| 81 | M | 38 | B-ALL | BCR-ABL |
| 82 | M | 15 | B-ALL | BCR-ABL |
| 83 | F | 50 | B-ALL | - |
| 84 | M | 19 | B-ALL | HOX11, EVI1 |
| 85 | M | 24 | B-ALL | - |
| 86 | M | 16 | B-ALL | BCR-ABL |
| 87 | F | 24 | B-ALL | - |
| 88 | M | 52 | B-ALL | MLL, HOX11, EVI1 |
| 89 | F | 44 | B-ALL | BCR-ABL |
| 90 | F | 26 | B-ALL | - |
| 91 | F | 12 | B-ALL | BCR-ABL |
| 92 | F | 58 | B-ALL | BCR-ABL |
| 93 | M | 13 | B-ALL | HOX11, EVI1 |
| 94 | M | 31 | B-ALL | HOX11, EVI1, MLL-AF4 |
| 95 | M | 12 | B-ALL | - |
| 96 | M | 22 | B-ALL | HOX11, EVI1, TEL-ABL |
| 97 | F | 22 | B-ALL | - |
| 98 | M | 8 | B-ALL | E2A-PBX1, EVI1 |
| 99 | F | 9 | B-ALL | EVI1 |
| 100 | M | 18 | B-ALL | - |
| 101 | F | 27 | B-ALL | BCR-ABL |
| 102 | F | 25 | B-ALL | - |
| 103 | M | 34 | B-ALL | BCR-ABL, MLL, HOX11, EVI1 |
| 104 | F | 9 | B-ALL | BCR-ABL, MLL-AF10, HOX11, EVI1 |
| 105 | M | 16 | B-ALL | MLL, HOX11, EVI1 |
| 106 | F | 48 | B-ALL | HOX11, EVI1 |
| 107 | F | 32 | B-ALL | TEL-AML1 |
| 108 | F | 41 | B-ALL | HOX11, EVI1, BCR-ABL |
| 109 | F | 23 | B-ALL | - |
| 110 | M | 7 | B-ALL | - |
| 111 | F | 6.1 | B-ALL | BCR-ABL |
| 112 | M | 4.1 | B-ALL | - |
| 113 | M | 4 | B-ALL | TCR-IgK |
| 114 | F | 2 | B-ALL | TCR-IGH/IGK, TEL-AML1 |
| 115 | F | 3 | B-ALL | TCR-IgH |
| 116 | M | 5 | B-ALL | - |
| 117 | M | 3 | B-ALL | - |
| 118 | M | 3 | B-ALL | TEL |
| 119 | M | 5 | B-ALL | TEL |
| 120 | M | 6.2 | B-ALL | TEL |
| 121 | M | 2 | B-ALL | - |
| 122 | M | 3 | B-ALL | TEL |
| 123 | M | 2 | B-ALL | MLL |
| 124 | F | 10 | B-ALL | IgH，FLT3-TKD |
| 125 | M | 4 | B-ALL | - |
| 126 | F | 12 | B-ALL | MDR1 |
| 127 | M | 3 | B-ALL | IgH |
| 128 | M | 3 | B-ALL | TCRγ |
| 129 | F | 4 | B-ALL | Tel, IgH, IgK, TCRγ，MDR |
| 130 | F | 10 | B-ALL | TEL，IgH，TCRγ |
| 131 | F | 3.3 | B-ALL | - |
| 132 | F | 2.9 | B-ALL | - |
| 133 | M | 7 | B-ALL | BCR-ABL |
| 134 | M | 4 | B-ALL | MDR, IgH, TCRγ |
| 135 | F | 5 | B-ALL | TEL |
| 136 | M | 2.7 | B-ALL | MDR1 |
| 137 | M | 12 | B-ALL | TCRβ, TCRγ |
| 138 | M | 2.5 | B-ALL | TEL |
| 139 | M | 9 | B-ALL | - |
| 140 | M | 3.3 | B-ALL | BCR-ABL |
| 141 | F | 7 | B-ALL | - |
| 142 | F | 3 | B-ALL | TEL |
| 143 | F | 10 | B-ALL | MLL |
| 144 | F | 1.4 | B-ALL | MLL |
| 145 | M | 2.3 | B-ALL | - |
| 146 | M | 7 | B-ALL | - |
| 147 | M | 2 | B-ALL | - |
| 148 | M | 4.7 | B-ALL | - |
| 149 | M | 10 | B-ALL | - |
| 150 | M | 13 | B-ALL | FLT3-ITD, TCRγ, IGH, IGK |
| 151 | M | 11 | B-ALL | IGH, IGK, MDr1, TCRγ, TCRβ. |
| 152 | F | 4.7 | B-ALL | - |
| 153 | F | 8 | B-ALL | TEL-AML1, IGH |
| 154 | F | 4 | B-ALL | TEL |
| 155 | M | 2.9 | B-ALL | IgH, FLT3-TKD |
| 156 | M | 7 | B-ALL | BCR-ABL, IGH |
| 157 | M | 9 | B-ALL | Tel，IgH，TCRγ. |
| 158 | M | 5 | B-ALL | BCR-ABL |
| 159 | M | 10 | B-ALL | TEL |
| 160 | M | 5 | B-ALL | IgH, TCR, MDR |
| 161 | M | 9 | B-ALL | IgH, TCR |
| 162 | F | 3.3 | B-ALL | - |
| 163 | F | 9 | B-ALL | E2A-PBX1 |
| 164 | M | 2 | B-ALL | - |
| 165 | F | 3.8 | B-ALL | TEL-AML1, TCRγ, IGH, IGK |
| 166 | F | 5 | B-ALL | IgH |
| 167 | F | 4 | B-ALL | TEL-AML1 |
| 168 | F | 5.6 | B-ALL | BCR-ABL |
| 169 | M | 3 | B-ALL | IgH |
| 170 | M | 5 | B-ALL | IgH,IgK，MDR |
| 171 | M | 8 | B-ALL | IgH, MDR |
| 172 | M | 6 | B-ALL | - |
| 173 | M | 1.6 | B-ALL | MLL |
| 174 | F | 1.9 | B-ALL | IgK |
| 175 | M | 4 | B-ALL | TCR-IgH |
| 176 | M | 5.7 | B-ALL | IgH, IgK, TCR |
| 177 | M | 7 | B-ALL | IgH, IgK, TCR |
| 178 | M | 6 | B-ALL | TEL |
| 179 | M | 1.5 | B-ALL | MDR1 |
| 180 | F | 7 | B-ALL | TEL-AML1, TCR, IGH, IGK |
| 181 | M | 3 | B-ALL | TCF3-PBX1 E2A(-) |
| 182 | M | 12 | B-ALL | BCR-ABL |
| 183 | M | 4 | B-ALL | - |
| 184 | F | 9 | B-ALL | Tel，TCR, IgH，MDR1 |
| 185 | M | 4 | B-ALL | Tel, IgK, TCR |
| 186 | F | 6 | B-ALL | TEL-AML1 |
| 187 | F | 6.1 | B-ALL | E2A, IgH |
| 188 | F | 12 | B-ALL | IgH |
| 189 | M | 2 | B-ALL | TEL |
| 190 | F | 12 | B-ALL | TCF3-PBX1, E2A |
| 191 | M | 42 | T-ALL | - |
| 192 | M | 26 | T-ALL | - |
| 193 | M | 32 | T-ALL | - |
| 194 | M | 13 | T-ALL | - |
| 195 | M | 23 | T-ALL | - |
| 196 | F | 19 | T-ALL | - |
| 197 | M | 40 | T-ALL | BCR-ABL |
| 198 | M | 27 | T-ALL | - |
| 199 | F | 33 | T-ALL | EVI1 |
| 200 | F | 34 | T-ALL | - |
| 201 | M | 25 | T-ALL | - |
| 202 | M | 74 | T-ALL | HOX11-EVI1 |
| 203 | M | 12 | T-ALL | - |
| 204 | M | 51 | T-ALL | - |
| 205 | M | 27 | T-ALL | HOX11-EVI1 |
| 206 | M | 12 | T-ALL | - |
| 207 | M | 16 | T-ALL | - |
| 208 | M | 41 | T-ALL | - |
| 209 | M | 15 | T-ALL | - |

Note: The non-fusion genes mean the abnormal expression of the related genes.
